# Qualitative exploration of the experiences and perceptions of diet in psoriasis management among UK adults

**DOI:** 10.1136/bmjopen-2024-085536

**Published:** 2025-04-09

**Authors:** Poppy Hawkins, Sarah Mason, Kate Earl, Thanasis G Tektonidis, Rosalind Fallaize

**Affiliations:** 1 Life and Medical Sciences, University of Hertfordshire, Hatfield, UK; 2 Department of Sport, Health Sciences and Social Work, Oxford Brookes University, Oxford, UK

**Keywords:** Psoriasis, Patient-Centered Care, NUTRITION & DIETETICS, DERMATOLOGY, QUALITATIVE RESEARCH

## Abstract

**Abstract:**

**Objective:**

This study aimed to explore the use, experiences and perceptions of diet in psoriasis management among adults with lived experience in the UK.

**Design:**

Qualitative. Data were analysed thematically using a reflexive thematic approach.

**Setting:**

Online discussions with adults living with psoriasis in the UK.

**Participants:**

Nine adults (two men, seven women) ≥18 years of age, living in the UK, English speaking, with a diagnosis of psoriasis of any severity.

**Results:**

Four key themes were generated: (1) impact of diet, (2) dietary modification, (3) dietary information and (4) dietary support. Overall, the majority (n=8) perceived that diet had an impact on their psoriasis. Most participants (n=7) reported trying restrictive diets including dairy free, gluten free and ‘cleanses’ to help manage their psoriasis with limited success. A perceived lack of dietary support resulted in participants relying on social media and online forums for dietary information. Participants reported a high cognitive burden due to the lack of reliable nutrition guidance and insufficient dietary support from healthcare professionals (HCPs).

**Conclusions:**

Participants rely on social media and online forums for dietary information, which suggest unsubstantiated restrictive diets that could negatively impact health. Participants felt overwhelmed by dietary recommendations and wanted more relevant dietary support. In the absence of evidence-based dietary information for psoriasis, HCPs need to be able to provide basic dietary support and combat misinformation. Larger studies aimed at understanding how best to support people with psoriasis are needed.

**STRENGTHS AND LIMITATIONS OF THIS STUDY**

- The qualitative design of the study allowed for in-depth exploration and rich insight into the participant’s experience.
- This study employed Braun and Clarke’s reflexive thematic analysis, a flexible approach that allowed researchers to effectively capture and represent participants’ reported experiences.
- Adherence to the Consolidated Criteria for Reporting Qualitative Research checklist enhanced the quality, rigour and transparency of the study.
- The study provides valuable insights, but the findings are based on a small homogeneous sample, which may limit generalisability.

## Introduction

 Psoriasis is a chronic, immune-mediated, inflammatory skin disease associated with arthritic, cardiovascular, metabolic and psychological comorbidities.^1 2^ There are an estimated 60 million people living with psoriasis (PLwP) globally, and in the UK, approximately 2% of the adult population are living with psoriasis.^3^ The chronic, painful and visible symptoms of the disease can have a substantial negative impact on quality of life (QoL).^4^

Research indicates that lifestyle can impact psoriasis symptoms.^5 6^ Reducing alcohol,^7^ limiting stress^8^ and smoking cessation^9^ have been shown to improve psoriasis symptoms. Furthermore, obesity is more common in PLwP compared with controls, and a higher body mass index is associated with increased psoriasis severity, attributed to adipose-driven inflammatory activity.^10^ Current evidence on the role of diet in the management of psoriasis is limited to weight loss in those living with overweight or obesity and a gluten-free diet (GFD) in those with coeliac disease or gluten sensitivity.^11 12^ There are no dietary guidelines for psoriasis, and there is high demand for information on diet from both healthcare professionals (HCPs) and PLwP. The question ‘Do lifestyle factors such as diet, dietary supplements, alcohol, smoking, weight loss and exercise play a part in treating psoriasis?’ was identified as the top research priority for psoriasis by the James Lind Alliance Priority Setting Partnership.^13^ However, research on the experiences of PLwP, including their use and perceptions of the role of diet, is scarce globally.^11^ Emerging data suggest that PLwP trial restrictive diets without guidance from an HCP, to try and help manage their psoriasis,^14^ which could lead to micronutrient deficiencies and negatively impact QoL.^15 16^ There are limited data on the practices of PLwP. Individuals with other skin conditions report using unregulated platforms including Instagram and online forums for nutritional advice to manage their condition.^17^

No studies in the UK have explored the use of diet in PLwP, their experiences of dietary modifications or sources they rely on in the absence of evidence-based dietary guidelines. With an estimated 1.1 million PLwP in the UK, this represents an important research gap. Exploring how PLwP use and perceive the impact of diet will play a key role in understanding the potential effect on both psoriasis and the health and well-being of PLwP, crucial for providing holistic care for patients. This study aims to explore in-depth the experiences and use of diet in psoriasis management among adults with lived experience in the UK through qualitative methods.

## Methods

Due to the scarcity of literature on this topic among a UK population, an explorative qualitative study was undertaken to enhance understanding and provide in-depth insights. Consolidated Criteria for Reporting Qualitative Research, a 32-item checklist for interviews and focus groups, was used to guide the reporting of the study findings.^18^

### Study design and participant recruitment

Qualitative semistructured interviews were conducted with UK adults with psoriasis. Participants were recruited online via Facebook. The study was posted in a private Facebook group for PLwP in the UK, which comprised over 15 000 members at the time of recruitment. Purposive sampling was employed to recruit participants. The eligibility criteria for this study were aged≥18 years, currently living in the UK, English speaking and a medical diagnosis of psoriasis of any severity. No incentive was advertised on the study recruitment post; however, following participation, participants were offered a £30 remuneration voucher for their time. Participant information and informed consent forms were emailed to each participant prior to undertaking the interview. At the start of the interview, the interviewer went through all forms and obtained verbal informed consent. Participants were aware that the research team wanted to explore the perceptions of PLwP on diet.

### Patient and public involvement

The design of this study was guided by the outcomes of previous cross-sectional questionnaires asking PLwP about their diet. The topic guide for the interviews in this study was informed by the responses given in these earlier studies. Patients were not involved in the study’s design, recruitment or completion. The results will be shared with the study participants and public through this publication.

### Data collection

Semi-structured individual interviews with UK adults with psoriasis were conducted to explore the perceived role of diet in the management of psoriasis. Topic guides were developed by RF and SM (both female). RF is a registered dietitian and Associate Professor in Research at the University of Hertfordshire, with extensive experience in qualitative research. SM was a final year dietetics student with an interest in diet and psoriasis. The topic guides were used to ensure interviews were consistent, but participants were also encouraged to expand on answers and express their opinions freely. The topic guide is provided as an online supplemental file. The interviewer asked participants to clarify answers and comments where meaning was unclear and frequently checked with participants whether their understanding of the meaning of their answers was correct. All interviews were conducted by one researcher (SM) with a single participant at a time. Each interview lasted approximately 1 hour, and all were conducted online via remote meeting applications, Microsoft Teams and Zoom. All interviews were audio recorded and transcribed verbatim. Data saturation guided the sample size; interviews were conducted until saturation was reached.

Psoriasis severity was self-reported by participants during interviews, as formal Psoriasis Area and Severity Index (PASI) scores or clinician assessments were not available. Participants were asked to describe their perceived severity, body surface area affected and were asked about any PASI scores provided by their healthcare providers, to ascertain severity scores based on mild, moderate and severe.^19^

### Data analysis

Data were analysed using a reflexive thematic approach based on the work of Braun and Clarke.^20^ The analysis process began with familiarisation with the data. The researchers PH and SM familiarised themselves with the data through immersion in the audio files and transcripts of the individual interviews of each participant. PH (female) is a PhD student and registered nutritionist, with experience in conducting qualitative research and thematic analysis. Subsequently, PH and SM independently coded the data using NVivo software. The codes reflected each researcher’s own interpretations of patterns and meaning throughout the dataset. PH and SM then independently generated themes for the dataset through organisation of their independent codes. All themes were then discussed together with the wider research team (all authors of this paper) to explore the interpretations of the data using a collaborative and reflexive approach. Through these discussions, four key themes were generated, which were divided into subthemes.

## Results

### Participants

17 individuals expressed interest in taking part, with 10 consenting to participate in the interviews. One participant dropped out prior to the interview with no disclosed reason. Data saturation was achieved during these interviews, meaning that data replication was observed, and no new themes or insights were generated from the interviews,^21^ and no further recruitment was deemed necessary. Overall, nine participants took part in the study (two males, seven females). The demographic information of the participants is summarised in table 1.

**Table 1:** Demographics and characteristics of study participants (n=9)

Variable	N
Sex	
Female	7
Male	2
Age, years: mean (range)	39 (25–53)
Psoriasis duration, years: mean (range)	17 (2–34)
Ethnicity	
White British	9
Medication (current)	
Topical steroids	5
Biologicals	3
Self-reported psoriasis severity	
Mild	2
Moderate	2
Severe	5

### Generated themes

**Figure 1:**
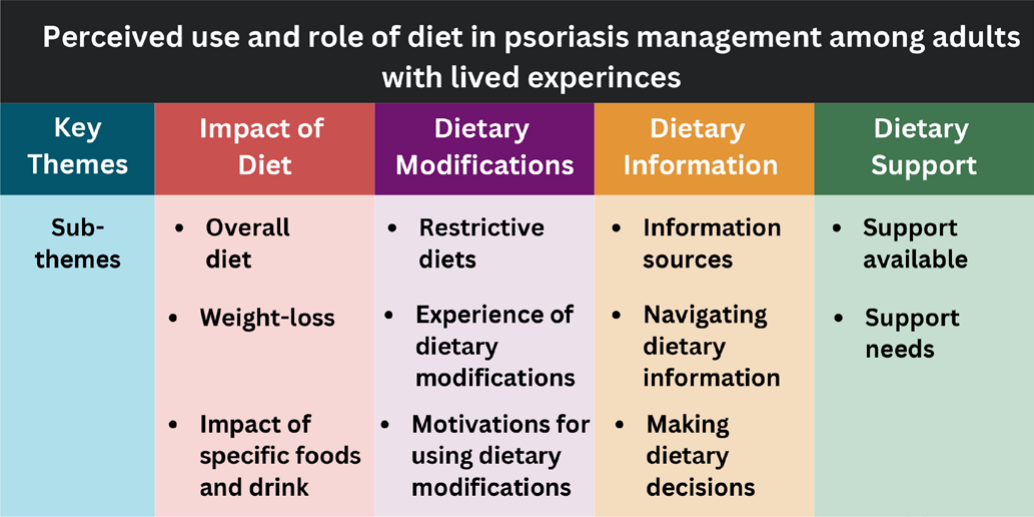
Key themes and subthemes generated during interviews with UK adults living with psoriasis, regarding the use and experience of dietary modifications.

Four key themes were generated from the collected data: (1) impact of diet, (2) dietary modification, (3) dietary information and (4) dietary support. Each key theme contained multiple subthemes, which are summarised in figure 1.

### Key theme 1: impact of diet

Participants discussed their thoughts on the role of diet in the management of psoriasis. They described the dietary factors that worsened or improved their psoriasis. A negative difference in psoriasis symptoms was more often experienced by participants than a positive difference to psoriasis symptoms through diet. Participants commonly described negative differences as a ‘flare’ or ‘flare-up’ which is an episode of worsened psoriasis symptoms. Additionally, participants also discussed increased itch, redness and drier skin when describing the negative differences that they experienced.

#### Subtheme: overall diet

All participants (n=9) believed that diet could play a role in the management of psoriasis, and most (n=8) reported that diet had impacted their psoriasis in some way. The majority (n=7) stated they notice a negative difference in their psoriasis when they do not eat a healthy, balanced diet.

…when I eat worse, it is worse. And when I eat healthy, it does get slightly better. [Recently] I haven't been eating very well and it is getting a lot worse than it used to be… (Participant 4)

I know that when I'm eating healthier, it is… it doesn't clear up, but it does fade and it is better. (Participant 9)

I don't need the Methotrexate anymore and I put so much down to not needing it because I've got a much better diet than I ever had. (Participant 2)

#### Subtheme: weight-loss

Regarding weight loss, one of the few evidence-based dietary recommendations for psoriasis, those whose weight had fluctuated in their adult life (n=4) reported mixed impact of weight loss or gain on their psoriasis.

…if I'm eating worse, I'm putting weight on - which then is making it worse. (Participant 4)

I haven't sort of, noticed that it’s [losing weight] made any difference, positive or negative on the psoriasis. (Participant 6)

#### Subtheme: impact of specific foods and drink

The majority (n=7) stated they notice a negative difference in their psoriasis when they eat certain ‘trigger’ foods. The most reported ‘trigger’ foods and drink for psoriasis were alcohol, dairy and sugar. Participants also reported that not eating enough fruit and vegetables had a negative impact on their psoriasis.

I guess the things we eat as well, do have an impact because there are times when I do eat some things and I seem to be more itchy with other things that don’t make me itchy. (Participant 8)

I don't eat anything that I know triggers [my psoriasis]. (Participant 3)

Dairy and alcohol are the big offenders [for making my psoriasis worse]. (Participant 5)

…if I have a lot of alcohol, that’s really bad. It takes… it’s not immediate - not like the next day but within the week. I know my… my face is a lot drier… it’s a lot redder. (Participant 9)

I've noticed that red wine will have a massive flare with me. (Participant 2)

…not eating enough fruit and vegetables… eating too much sugar [are dietary triggers for my psoriasis]*.* (Participant 4)

### Key theme 2: dietary modifications

Dietary modifications involved intentional changes in the food and drinks consumed. Dietary modifications were commonly discussed by participants alongside the impact these had on their psoriasis symptoms. Almost all of the dietary modifications were restrictive. Only one participant had tried adding supplements, but found they had no impact on their psoriasis.

primrose oil was one thing that was suggested and cod liver oil. So, I did start taking both of those, um… but they didn’t have an effect. (Participant 9)

#### Subtheme: restrictive diets

Most participants (n=7) had tried following at least one restrictive diet to try and help their psoriasis symptoms. A restrictive diet refers to an eating pattern that reduces or cuts out certain foods, food groups or energy intake. The most common restrictive diets tried by participants were reducing or removing dairy, cutting out nightshades and following GFDs. Nightshades are plants from the *Solanaceae* family, which include potatoes, tomatoes, peppers and aubergines.^14^ Dietary ‘cleanses’ were mentioned and involved numerous different restrictions. The ‘cleanses’ that participants attempted in this study were typically highly restrictive, involving either the consumption of only specific types of foods or juice-based diets, where participants drank only juice and water for a set number of days.

I've not had any dairy at all… [for 3 months]” and “[I tried going] completely gluten-free for three months (Participant 1)

[I am] actively avoiding nightshades (Participant 2)

I've done lots of cleansing diets… (Participant 5)

[I tried a diet] where you had to eat […] just apples for like 2 weeks. And that was supposed to be some, like sort of… cleanse. (Participant 6)

Participants reported mixed results from restricting dairy. Two of the four participants who had tried reducing or removing dairy reported no difference in symptoms, and two reported an alleviation of psoriasis symptoms. Weight loss was also reported as a consequence of following a dairy-free diet.

I found that cutting out milk made a bit of a [positive] impact (Participant 9)

I have noticed that I've lost weight during the dairy-free diet because obviously… you can’t eat so many things. (Participant 1)

Following a GFD and avoiding nightshades were frequently reported to have no impact on psoriasis symptoms by those who had tried these, and following overly restrictive low-fat diets were believed to have worsened one participants’ psoriasis.

I did follow it for quite a while and was having like, gluten-free bread and other things …] I didn't see a difference. (Participant 9)

Tried a gluten free diet for three months. And it made absolutely no difference whatsoever. And when I went back to eating copious amounts of gluten again, I didn't notice it got worse either. (Participant 1)

[I tried] nightshades… trying to avoid them. Um, and… but I didn't find that it worked (Participant 6)

When I was restricted and going down the complete healthy route of eating like, healthy as in eating disordered healthy - it would be low-fat this, low-fat that. Like completely skinny milk - and that’s when my skin was the worst. (Participant 2)

#### Subtheme: experience of dietary modifications

Those who had tried following a specific dietary modification to help their psoriasis frequently reported that it was difficult to adhere to (n=6). Preparing separate meals was a barrier for those who cooked for others, and cutting out foods or whole food groups meant that it was difficult to know what to replace them with. Restrictive diets are also reported to provide little enjoyment and limited reward. Participants often reported that the restrictive diets had not made any or much difference to their psoriasis symptoms, which was demotivating, or they had not been able to keep following them due to some requiring extremely strict exclusions.

I just think I don't want to make myself miserable either. And to take over my life to that extent, either without really thinking that that would work. And there’s a huge commitment, especially when you then cook for a family of five too… I don't want to take it out of their diets, so it will be quite difficult to do. (Participant 1)

I'm quite happy to kind of - give up things and try new things. And I'm… but yeah, I find I find it really hard to kind of… because when you've got kids and you're like, doing different meals and different… all the different things, so it is difficult to sustain. That’s… that’s the thing. I think I'm happy to do it for a week or two but then life gets in the way (Participant 9)

…I had a lot of dairy. So, it’s a lot to cut out. (Participant 1)

#### Subtheme: motivations for using dietary modifications

The main reason that participants reported wanting to try dietary modifications for their psoriasis were: (1) wanting a natural way to help manage the condition (n=4), (2) as a potential way to avoid starting or going back on medication that was perceived to be strong or associated with undesirable side effects (eg, immunosuppressants) (n=4) and (3) having autonomy and a sense of empowerment by being able to do something to help themselves, rather than being completely reliant on referrals to HCPs and dermatologists (n=5).

I’ve [always] looked for the more natural ways to control it. (Participant 5)

I'm so reluctant to go on something as strong as Methotrexate I have tried a gluten free diet for three months. (Participant 1)

I'm always looking for ways in which I myself can help the condition without always being referred … If there’s anything… natural ways that it can be better, then I'm always up for doing it that way. (Participant 8)

### Key theme 3: dietary information

Participants discussed where they obtained dietary information from, their experiences of navigating dietary recommendations from different sources and gave insights into the factors that influenced their decisions to make certain dietary modifications.

#### Subtheme: information sources

The participants frequently reported that online patient forums and groups, as well as social media and Google, were their main source of dietary information for psoriasis (n=8). This was primarily due to a lack of information from trusted sources, such as HCPs and organisations, and a lack of evidence-based information readily available to them. The dietary modifications recommended online and in the patient forums and social media groups were often restrictive. The restrictive dietary modifications often recommend eliminating certain food groups or specific foods and drinks from the diet, and often had strict rules on what can and cannot be eaten.

…every diet you could possibly suggest. No nightshades, no gluten, no meat, no red meat, no sugar, no dairy and no alcohol, and pretty much any combination of those diets suggested on forums and trialled by forum members]. (Participant 1)

If you listen to this doctor [an American doctor found online], I might as well just not eat because he’s going, “Nightshades, milk, cheese…” and I'm thinking, “Well, what can I eat?” “Chicken.” That was all what he was saying. And I thought, “No.” I couldn't live like that. I couldn't live with cutting all of them things out. (Participant 7)

However, participants also frequently reported that the online psoriasis forums were useful for general support, even if the dietary information was perceived by some to be misleading or unfounded. They reported that the groups gave them a chance to feel understood, a place to ask questions about psoriasis without judgement and hearing coping strategies other PLwP had used. Overall, the online groups were perceived in a positive light and provided participants with psychosocial support.

…it’s good to be around… or have access to people who have gone through the same kind of things. (Participant 6)

I find it helpful to know that there’s other people that are going through similar things to what I've done in the past […] but there’s a lot of mis-led information out there as well. (Participant 2)

#### Subtheme: navigating dietary information

Participants often reported that when looking for dietary advice, they felt overwhelmed by the amount of information available (n=7). The information was often contradictory and went against their better judgement. This caused uncertainty and added to the cognitive burden of participants, alongside resulting in them trialling diets even when they were sceptical about the reliability and health impacts of the dietary changes recommended.

… there are too many websites and too many pages with too many different conflicting things on and I get mind boggled [……] one will say, don’t eat that and then one will say, do eat that. (Participant 7)

…as soon as you put in Google [diet and psoriasis] all this information comes out and… You'll try anything. (Participant 7)

You’re not meant to remove whole food groups… […] So, I kind of question whether that is a good move. (Participant 1)

#### Subtheme: making dietary decisions

Participants reported that their decisions to try certain dietary modifications were influenced by before and after photos posted online by forum members who had changed their diets, anecdotal experience of dietary changes of other PLwP and popular wellness figures, even when they were sceptical (n=6). Participants reported trying dietary changes just to see if it would work for them, like it had for other people they had seen online.

[Those who] post pictures of before and after, who’d done the Hannah Sillitoe diet. And that is, I think, probably what made me do this dairy-free diet and if I'm honest, that is probably from seeing the difference in her skin on the pictures. I don't know her… I've never had a conversation with her, but just thinking… if, if that is real, then I’d be silly to keep my mind closed to that as well. So, I’m willing to try it. Um, so… so it does influence me even if I take it with a pinch of salt. (Participant 1)

I've been following, you know, Hannah Sillitoe who cleared her psoriasis by having a very vegan healthy lifestyle? And it’s one of the reasons why I've become a vegan. (Participant 9)

### Key theme 4: dietary support

Participants discussed their experiences of the dietary support they had sought and received.

#### Subtheme: support available

All participants (n=9) perceived there to be a lack of dietary support available to them and that HCPs were reluctant to discuss diet during appointments. Participants recognised that this may be due to a lack of evidence of a relationship between diet and psoriasis. However, the lack of discussion about anything to do with diet was deemed unhelpful and left participants feeling frustrated. Additionally, if dietary advice was given by HCPs, it was reported to be vague healthy diet or weight-loss suggestions, without any specific information or support. This was perceived to be unhelpful and presumed dietary knowledge.

[The Doctor] just kept on saying, “Oh, there’s no cure. There’s nothing that you can do.” (Participant 9)

[when trying to discuss diet with HCPS] “they're a little bit nervous. Um, I don’t think they ever like to comment […] they’re very much like, um, “Just keep it varied. (Participant 2)

I think that expecting people to have like, a good knowledge of food, and what things can be replaced with, is… it’s just quite unfair. (Participant 3)

…dermatology and rheumatology, have both told me to, sort of lose weight and that'll help with the psoriasis and the psoriatic arthritis. But, um that’s it… and I haven't noticed any improvement. *(Participant 6)*

#### Subtheme: dietary support needs

Most participants (n=8) stated that they would benefit from dietary support from an HCP with nutritional expertise, to help them navigate the overwhelming amount of often contradictory dietary advice available and the cognitive burden of trying to decide what was safe. Participants stressed the importance of evidence-based advice. Recognising that although there was limited evidence on the relationship between diet and psoriasis, they were still willing to try diets suggested online, by friends and family or from popular wellness figures in case they did work for them. As a result, they wanted support to be able to try these diets safely and better understand the potential health implications, particularly for elimination diets.

Just knowing what is safe, what will be a good move, where I can start… (Participant 8)

… I didn't know how to do it properly. Like I don't know what gluten’s in […] it’s everywhere, isn’t it? (Participant 2)

Because such huge amounts of your food groups you’re cutting out, I think I’d want to know that I was not depriving myself. (Participant 1)

…when someone’s expecting you to cut something… that might make up quite a big part of your diet, then you need to know what you can be using instead. (Participant 3)

There was no observed difference in perception or experience across any demographic characteristics. However, the sample size was too small to conduct any comparative analysis, and the sample size may also be the reason that no differences were observed.

## Discussion

Despite a growing interest in the role of diet in psoriasis management, there has been limited research exploring the perceptions of PLwP on the use and role of diet. To the best of our knowledge, this is the first study to explore this topic in PLwP in the UK. The findings of this study have identified the challenges PLwP face and have highlighted potential gaps in support for PLwP regarding diet, alongside areas for further research to improve psoriasis care.

Most participants in this study perceived that diet had an impact on their psoriasis and took proactive measures to avoid foods known to trigger psoriasis flare-ups or worsen symptoms. The most common dietary triggers perceived to negatively affect psoriasis in this study were alcohol, dairy and sugar. This mirrors findings from previous studies.^14^ Additionally, participants also perceived that eating a ‘generally healthy diet’ characterised by consuming plenty of fruit and vegetables had a positive impact on their psoriasis. Previous studies have found that fruit and vegetable consumption was reported to alleviate symptoms by PLwP^22^ and that higher fruit and vegetable intake was associated with lower psoriasis severity.^23 24^

Specific diets were also commonly trialled by participants to try and help manage their psoriasis, all of which were restrictive. The most trialled diets were dairy restriction, gluten free, avoiding nightshades and a range of different cleanses, with limited or no perceived impact on psoriasis symptoms.

Dairy restriction was reported as a dietary modification trialled by four of the participants in this study, with mixed results. Previous research has reported that dairy elimination or restriction is common in PLwP in the US and provided alleviation of psoriasis symptoms in almost 50% of people who removed it.^14^ However, there is limited research investigating the impact of dairy consumption on psoriasis severity. The reasoning behind eliminating dairy may be attributed to concerns about the proinflammatory effect of saturated fat, which is high in certain dairy products.^25^ However, research indicates that dairy may have neutral to favourable effects on inflammation.^25^ Furthermore, low-fat fermented dairy products such as yoghurt have been shown to have anti-inflammatory effects attributed to the presence of probiotics.^25 26^ Dairy products are also key sources of high-quality protein and essential micronutrients, including vitamin B12, calcium, magnesium and zinc.^27^ Eliminating or restricting dairy may negatively impact the intake of essential nutrients.^28^ There is an absence of research on the type of dairy product perceived to have a negative impact on psoriasis symptoms, and this warrants further investigation. Additionally, one of the participants who reported removing dairy also reported losing weight while following a dairy-free diet. Weight loss in PLwP who are also living with obesity or overweight has been shown to improve psoriasis symptoms.^12^

Avoiding nightshades was a common dietary modification reported by participants. Nightshades are a family of plants that include potatoes, tomatoes, peppers and aubergines. They contain solanine and alkaloids which have been linked to inflammation in mouse models.^14 29^ However, no human studies support this association; furthermore, nightshades are high in fibre and a rich source of antioxidants. Additionally, participants in this study and others have reported that eating fruit and vegetables improved psoriasis symptoms.^23 24^

GFDs were also commonly trialled by PLwP, and research suggests that they could alleviate psoriasis symptoms, but only in PLwP who have coeliac disease or a sensitivity to gluten; otherwise, it is not recommended.^12^ Previous research has also indicated that PLwP trial a GFD with mixed effects.^14^ It is unclear whether PLwP recognise that the evidence only suggests following a GFD for those PLwP who are coeliac or have a diagnosed gluten sensitivity. Psoriasis is associated with numerous other autoimmune diseases, including coeliac disease.^30^ However, greater awareness may be needed regarding who this type of diet is appropriate for, as GFDs have been shown to be low in dietary fibre.^31^ Greater dietary fibre intake is associated with a lower risk of cardiovascular disease and coronary heart disease, as well as lower systemic inflammation.^32 33^ Dietary fibre also has appetite-regulating and antiobesogenic properties.^32^ This is relevant to PLwP considering the associated comorbidities.

The combination of the prebiotic properties of dietary fibre consumption, alongside the probiotics found in fermented dairy products, may exert a moderating influence on the pathogenesis of psoriasis,^34^ by promoting gut health and subsequently regulating the innate and adaptive immune responses.^34^ Therefore, the health benefits of these commonly eliminated foods are important considerations, as well as understanding the substitutions that may be consumed in place of the eliminated foods. Most participants felt overwhelmed with the number of dietary recommendations available online and did not feel as though they had the knowledge to be able to navigate them safely. This led to people trying restrictive diets, often against their better judgement and without the knowledge of how to do so safely. Following restrictive diets without the guidance of an HCP can lead to micronutrient deficiencies,^15^ and studies show that individuals who follow restrictive diets report significantly lower QoL and negatively impact mental well-being.^35^ The restrictive dietary practices that PLwP adopt could therefore have detrimental impacts on both physical and mental health. To build on the findings of this study, future research should further investigate the diets commonly trialled by PLwP to better understand the potential impacts of these on both physical and mental health.

This study found that there was a perceived lack of dietary support available for PLwP from HCPs. Despite recognising the shortage of evidence-based information on diet and psoriasis, participants often felt that HCPs were reluctant to discuss diet at all, and if dietary information was given, it was perceived to be vague and lacking useful instruction. This led to individuals seeking dietary advice from alternative sources, primarily wellness figures and other PLwP on online forums or social media. The main motivations for participants wanting to trial dietary modifications were to find a natural way to help manage the condition, avoid medication side effects and wanting autonomy over their condition. These findings echo previous studies which found that PLwP mainly use complementary or alternatives to conventional medication due to treatment failures or unwanted side effects.^36^ Previous research has highlighted the amount of dietary misinformation on social media,^37^ which further highlights the importance of providing dietary support to this group.

Moreover, a recent study exploring dermatology professionals’ experiences of dietary habits of outpatients (n=159) found that psoriasis patients were one of the patient groups reported to ask about nutrition most often.^38^ However, 73.1% of dermatologists did not feel confident in answering these questions, and over 90% felt that additional nutrition training and access to specialist dietician support would be of benefit to dermatology practice.^38^ This suggests that not only is there a high demand for dietary support patients but also that HCPs may require further training and resources to be able to provide this type of support. Considering patients’ values and preferences alongside their physical, social and emotional needs is a core part of patient‐centred care.^39^ All of which further highlights the need for research in this area. Furthermore, many of the psoriasis-associated comorbidities are widely recognised to be related to diet.^33 40^ As a result, engaging in discussions about dietary considerations with HCPs with nutritional expertise, or having access to evidence-based dietary support, could improve comprehensive care for PLwP. While also lessening the reliance on unsubstantiated online sources for dietary information.

### Limitations

The small sample size of this study, which comprised all white British and predominantly female participants means that further research is required to establish whether these findings are generalisable to PLwP across the UK. Furthermore, participants were recruited via an online psoriasis support group, which could have influenced the answers given by participants regarding sources of dietary information. The topic of the study may have led to a sample that perceived there to be a role for diet in managing psoriasis. Additionally, all dietary information and impact on psoriasis was self-reported. However, this was an initial exploratory study into a previously unexplored population, and despite the limitations of this study, the findings provide novel and in-depth insight into the experience of PLwP regarding diet and potential support gaps in psoriasis care, which has the potential to inform subsequent larger research studies.

## Conclusion

PLwP feel overwhelmed with the number of dietary recommendations claiming to help psoriasis and require more support to be able to navigate them. From the patient perspective, current dietary support provided by HCPs is lacking. As a result, PLwP turn to unregulated online platforms. This could have detrimental implications on the health and well-being of PLwP and therefore HCPs need to be able to confidently discuss diet and provide basic dietary support to PLwP until evidence-based dietary guidance for psoriasis is available. Understanding dietary support needs in psoriasis care from an HCP perspective warrants further investigation. The findings of this exploratory qualitative study will inform larger quantitative investigations of dietary practices of PLwP in the UK. This will enable better understanding of the use of diet, dietary support needs and opportunities to provide tailored support.

## Supplementary material

10.1136/bmjopen-2024-085536 online supplemental file 1

## Data Availability

No data are available.
