# Supplementary material for: Qualitative exploration of the experiences and perceptions of diet in psoriasis management among UK adults
Source: BMJ Open. 2025 Apr 9;15(4):e085536. doi: 10.1136/bmjopen-2024-085536 (PMC11987128; doi:10.1136/bmjopen-2024-085536)
Supplement: online supplemental file 1 [file bmjopen-15-4-s001.docx]

## **Topic Guide for Individual Interviews**

**INTRODUCTION**

Thank you for agreeing to take part in our study on perceptions and experiences of nutrition in the progression and management of psoriasis. The study aims to explore to what extent individuals with psoriasis perceive that diet is important and/or plays a role in the development/progression and management of their condition.

We will ask some general health background questions first then specific questions about your perceptions and experience of the impact of nutrition in the progression (e.g., flares) and management of your plaque psoriasis. The interview will last around 45 minutes or longer, but it depends how much you have to say. If we ask something that’s not relevant just let us know and we will move on. We are really interested in your experiences so feel free to give us as much detail as you’d like to. When we analyse all the interviews, we will write up the findings without referring to your name or anything that identifies others in your household or family.

So that we can accurately type up what you say today I’d like to audio record the interview. The recording will be deleted once the transcript has been written up. Is that OK with you?

Do you have any questions before we start?

**Confirm answers have been understood and recorded correctly by checking with participants. Ask participants to expand if needed**.

**PART A: Demographics**

Age of participant

Do you have psoriasis?

If yes, what type, & how severe?

Use PASI Index

How long have you had psoriasis/ when were you diagnosed?

Did anything trigger your initial symptoms/diagnosis? (i.e., Infection/illness?)

Have you noticed any pattern in your symptoms?

**PART B: ROLE OF DIET IN MANAGING PSORIASIS**

What factors do you think contribute most to your **flare ups and/or condition (i.e., development)**?

Does stress impact your condition?

How would you describe your stress levels?

Does what you eat and/or drink impact your condition?

Have you ever suffered from pain/wind/bloating?

How frequent are your bowel movements?

Are your bowel movements often soft and/or hard and difficult to pass?

Do you think family history and/or genetics impacts your condition?

Does Illness or infection, including testing positive for Covid-19, impact your condition?

Do you think weight impacts your psoriasis?

Do other conditions and/or co-morbidities impact your condition?

What factors do you think play a role in **managing a flare up and/or your condition**?

*(As above for probing about known factors – including medication)*

Have you identified any dietary triggers related to your condition?

What are these?

How did you identify these? (e.g., using a symptoms diary)

Did you and/or are you still changing your diet are a result of this?

Have you ever **excluded** anything from your diet?

What did you exclude?

Was this helpful/ beneficial and in what way?

CHECK if related to another condition

Did you and/or are you still changing your diet are a result of this?

Have **you ever added** anything to your diet?

What did you exclude?

Was this helpful/ beneficial and in what way?

CHECK if related to another condition

Did you and/or are you still changing your diet are a result of this?

Have you received any dietary advice in relation to managing your psoriasis?

From whom?

Did you follow it?

How useful was it?

Which lifestyle/dietary factors were targeted by the person who gave you advice? [If mentioned prompt for weight re: weight stigma]

Would you consider making changes to your diet in the future?

If not tried previously, what are the barriers that have prevented you from making dietary changes? (E.g., lack of evidence, cooking skills, motivation etc.)

What support would be useful to help you make changes to your diet? (If perceived as useful/beneficial)

Are you aware of anyone that has successfully managed their psoriasis with diet?

**PART C: CURRENT DIETARY HABITS**

How would you describe your diet? (Healthy/unhealthy? Vegetarian? Cooked/convenience? Restrictive? Varied? Ketogenic? Carnivorous?)

Do you drink alcohol? (If so, what pattern? How many units? What type?)

**PART C: PERCEPTIONS OF RECEIVING NUTRITION ADVICE**

Are you a member of any patient support groups? If so, which do you find most helpful & for what reasons?

Re: diet, do you think it would be beneficial to have access to a dietitian and/or nutritionist as part of your management? (I.e., freely available on the NHS).

**THAT’S THE END OF THE INTERVIEW, thank you for your time. Is there anything else you’d like to tell me or add?**
