# Supplementary material for: Qualitative exploration of the experiences and perceptions of diet in psoriasis management among UK adults
Source: BMJ Open. 2025 Apr 9;15(4):e085536. doi: 10.1136/bmjopen-2024-085536 (PMC11987128; doi:10.1136/bmjopen-2024-085536)
Supplement: Supplementary file 2 [file bmjopen-2024-085536.reviewer_comments.pdf]

PEER REVIEW HISTORY

BMJ Open publishes all reviews undertaken for accepted manuscripts. Reviewers are asked to complete a checklist review form and are provided with free text boxes to elaborate on their assessment. These free text comments are reproduced below.

ARTICLE DETAILS

Title (Provisional)

A QUALITATIVE EXPLORATION OF THE EXPERIENCES AND PERCEPTIONS OF DIET IN PSORIASIS MANAGEMENT AMONG UK ADULTS

Authors

Hawkins, Poppy; Mason, Sarah; Earl, Kate; Tektonidis , Athanasios G; Fallaize, Rosalind

---

VERSION 1 - REVIEW

**Reviewer**	**1**
**Name**	**Maruthappu, Thiviyani**
**Affiliation**	**King's College London, Nutritional Science**
**Date**	**25-Jun-2024**
**COI**	**Honrorarium received from various pharmaceutical companies for invited speakers**

This is an interesting and well written paper highlighting the need for further work and support in a key area of research.

Introduction - the introduction should include reference to our current understanding of the role of diet and psoriasis for example the influence of body weight and adiposity. For example Zanesco et al., 2022, Nutrition Bulletin. At present body weight represents a driving factor in psoriasis severity - were participants asked about this and what had they been told by healthcare professionals on the impact of BMI?

It is also worthwhile mentioning that the role of diet was the number 1 question in the Priority Setting Partnership by the James Lind Alliance indicating that this is a significant unmet need for people who live with the disease

There could also be greater information on the existing evidence for the impact of dietary modifications on mental health and well being.

Were mental health questionnaires e.g. DLQI score used as part of data collection? It would be interesting to note if higher DLQI scores were associated with more restrictive practices?

There is no mention on dietary modifications to improve Gut Health - is this something that participants mentioned? This would be worth expanding on if so.

Please explain more clearly what a cleanse may entail - this is a rather general term

How did participants self-report psoriasis survey - were any validated tools use to assess this?

P9, Line 42 - please define nightshades early on as readers may not be aware of this family

P13, Line 56 please define restrictive diets - there are examples on the negative impact of restrictive diets on mental health that can be included.

**Reviewer**	**2**
**Name**	**Goni, Leticia**
**Affiliation**	**University of Navarra**
**Date**	**01-Jul-2024**
**COI**	**There is no competing interest**

This is an interesting study in which the authors analyze the experiences and use of diet in patients living with psoriasis. While this an interesting approaching there are major and minor comments that should be addressed via revisions or with additional clarifying information.

- In the abstract, please provide the aim of the study.
- In the introduction:
    - Include at least a reference for the sentence "However, there is limited evidence on the role of diet on the management of psoriasis (…)". In this sentence, you also confirm that there are no evidence-based dietary guidelines for psoriasis, but I searched the internet and found the National Psoriasis Foundation Medical Board dietary recommendations published in JAMA dermatology in 2018 (https://pubmed.ncbi.nlm.nih.gov/29926091/). Please check if there are any other guidelines and adapt the sentence.
    - You mention that there is no research on the experiences of patients living with psoriasis in the UK, but what about other countries? The introduction needs to include a clearer justification, including a review of studies in other countries.
- In the methods section:

- Of the 15000 Facebook members, how many were interested in taking part in the study? Of these, how many did not meet the inclusion criteria? o In terms of inclusion criteria, no maximum age limit was set for participation.
- How do you estimate the sample size?
- As the "topic guides" that you used in the interviews, could you provide them? At least the headings.

- In the results section:
  - In the first key theme you provide the frequency of participants who believed, who reported… but not in the rest of the themes. Please provide the number of participants who responds each idea or issue.
  - At the end you mention that no difference was observed by age groups, severities or sex, but what about the psoriasis duration?
- General comments:
  - Please check carefully abbreviations. For example, at the end of the second paragraph of the introduction you did not use the abbreviation for quality of life, or in the third paragraph of the methods section you did not use the abbreviation for people living with psoriasis.

Reviewer 3
Name Ballester-Martinez, María Asunción
Affiliation Hospital Universitario Ramon y Cajal, Dermatology
Date 02-Jul-2024
COI none regarding this article

This is an interesting article regarding dietary modifications in patients with psoriasis. Although exploratory, it includes a very low number of participants, probably not representative of people living with psoriasis (mostly women, 25-53 years, all white; education level not explained; none with conventional systemic treatment (although during the interviews methotrexate is mentioned several times); unknown history of comorbidities or previous dietary interventions; unexplained previous or normal dietary habits; all recruited through Facebook...) Those aspects are partially explained as limitations, but they make the study less generalisable. Besides, the content of the interviews is not sufficiently

explained. It is indicated that they are semi-structured interviews, but the specific content is not specified, which makes the study difficult to reproduce. Supplementary surveys asking about specific data on dietary habits and modifications would be of use to make the study more complete. It could be also interesting to analyse data taking into consideration if the participant had other comorbidities, which can also have an impact on patient´s diet. This aspect has not been addressed either. As for the results, only some objective results are provided. Although the direct quotations are interesting, they do not provide information that is as relevant for the reader. A larger study including more patients with more diverse profiles and quantitative aspects could make the study more complete.

**Reviewer**	**4**
**Name**	**Mitchell, Charlayne**
**Affiliation**	**Northwestern University**
**Date**	**03-Sep-2024**
**COI**	**N/A**

Overall, it is a nicely written manuscript. I did have three suggestions first if you decide to increase the size of the study for diversity you should read Hagaman and Wutich, "How Many Interviews Are Enough to Identify Metathemes in Multisited and Cross-cultural Research? Another Perspective on Guest, Bunce, and Johnson’s (2006) Landmark Study." Second, I wanted to ask if, on page 6, line 6 under the "method" section "The Consolidated criteria for reporting qualitative research (COREQ) should be capitalized as follows: Consolidated Criteria for Reporting Qualitative Research. Third, I am not sure if there is a transcription error or if Participant 1 misspelled "red meat" as "read meat" in the quote inserted on line 6 page 14 under "Key Theme 3: Dietary information."

Again, nicely written.

VERSION 1 - AUTHOR RESPONSE

Reviewer: 1
Dr. Thiviyani Maruthappu, King's College London
Comments to the Author: This is an interesting and well written paper highlighting the need for further work and support in a key area of research.

The authors would like to thank the reviewer for their comments and feedback, which have been addressed below.

1. Introduction - the introduction should include reference to our current understanding of the role of diet and psoriasis for example the influence of body weight and adiposity. For example Zanesco et al., 2022, Nutrition Bulletin. At present body weight represents a driving

factor in psoriasis severity - were participants asked about this and what had they been told by healthcare professionals on the impact of BMI?

Response: This information has been added to the introduction, with a citation to this reference – see line 81-83. Regarding weight-loss, those who had lost or gained weight were asked whether they had noticed any changes in psoriasis as a result - impact of weight changes were mixed. This has been highlighted in Theme 1: Impact of diets, in the results – see lines 200-201. Regarding information from healthcare professionals, weight-loss was vaguely suggested to one participant, but others had not received any information on BMI or weight-loss impact on psoriasis. This has been highlighted in Theme 4: dietary support, in the results – see lines 389-390 and 399-401.

2. It is also worthwhile mentioning that the role of diet was the number 1 question in the Priority Setting Partnership by the James Lind Alliance indicating that this is a significant unmet need for people who live with the disease

Response: This has been added to the introduction, with a citation to the James Lind Alliance PSP - see lines 86-89.

3. There could also be greater information on the existing evidence for the impact of dietary modifications on mental health and well-being.

Response: Further information on this has been added to the discussion of the paper - see lines 489-491.

4. Were mental health questionnaires e.g. DLQI score used as part of data collection? It would be interesting to note if higher DLQI scores were associated with more restrictive practices?
There is no mention on dietary modifications to improve Gut Health - is this something that participants mentioned? This would be worth expanding on if so.

Response: DLQI was not used as part of data collection in this study. This would have provided interesting insight into the impact of restrictive dietary modifications made by PLwP and has been included in the limitations of the study. However, the aim of this study was to gain an initial understanding of if and how people with psoriasis in the UK use diet. The authors have noted that understanding the dietary changes made would help improve understanding of the potential impact of dietary changes, specifically restrictive ones, on physical and mental health should be considered in future research to build on the findings of this study – see lines 484-489. Gut health was not mentioned by any participants nor the use of pre/probiotics so it was not mentioned in the results of the paper.

5. Please explain more clearly what a cleanse may entail - this is a rather general term

Response: An explanation of the types of cleanses that participants mentioned has been included in the results section, where the term “cleanse” is mentioned - see lines 237-240.

6. How did participants self-report psoriasis survey - were any validated tools use to assess this?
Response: Psoriasis severity was self-reported; no validated tools or clinician assessments were used. Participants were asked to describe their perceived severity, body surface area affected, and were asked about any PASI scores provided by their healthcare providers, to ascertain severity scores based on mild, moderate and severe. This has been made explicit in the methods, and self-reporting highlighted as a limitation - see lines 144-146 and 523.

7. P9, Line 42 - please define nightshades early on as readers may not be aware of this family

Response: A definition has been included in the results section, where the term "nightshades" is mentioned - see lines 236-237.

8. P13, Line 56 please define restrictive diets - there are examples on the negative impact of restrictive diets on mental health that can be included.

Response: A definition has been included in the suggested section, where the term "restrictive" is mentioned - see lines 233-234.

Reviewer: 2
Dr. Leticia Goni, University of Navarra
Comments to the Author: This is an interesting study in which the authors analyze the experiences and use of diet in patients living with psoriasis. While this an interesting approaching there are major and minor comments that should be addressed via revisions or with additional clarifying information.

The authors would like to thank the reviewer for their comments and feedback. Our revisions are described below.

1. In the abstract, please provide the aim of the study.

Response: A clear aim has been added to the abstract – see lines 21-22.

2. In the introduction:

o Include at least a reference for the sentence "However, there is limited evidence on the role of diet on the management of psoriasis (…)". In this sentence, you also confirm that there are no evidence-based dietary guidelines for psoriasis, but I searched the internet and found the National Psoriasis Foundation Medical Board dietary recommendations published in JAMA dermatology in 2018 (https://pubmed.ncbi.nlm.nih.gov/29926091/). Please check if there are any other guidelines and adapt the sentence.

Response: The sentence has been adapted, and a reference has been added for the above suggestion - see line 85. Regarding the term dietary guidelines, we are referring to official recommendations, issued by government bodies or health organisations, based on extensive nutritional research. Dietary guidelines should provide clear, evidence-based advice on what people should eat to maintain health and prevent/manage disease, and are specific whole dietary patterns. Dietary recommendations are generally not supported by large-scale studies or there may only be evidence for several recommendations but not for a specific dietary pattern for the specific disease. For psoriasis there are no specific dietary guidelines. The 2018 paper you reference (Ford et al., 2018 JAMA) is a systematic review that does suggest a low-calorie diet for weight loss in people living with obesity or overweight, for psoriasis, but ultimately the paper concludes that there is limited evidence on the role of diet in the management of psoriasis, and therefore not enough evidence for specific dietary guidelines. Thus, evidence is limited and does not provide dietary guidelines; it provides an overview of the evidence of diet and psoriasis and makes a recommendation for people living with psoriasis and obesity/overweight. This is reflected in the introduction of the study.

3. You mention that there is no research on the experiences of patients living with psoriasis in the UK, but what about other countries? The introduction needs to include a clearer justification, including a review of studies in other countries.

Response: A more explicit insight into the limited research conducted on the experiences of people living with psoriasis (PLwP) globally has been included in the introduction– further highlighting the rationale for exploration into this in a UK population of PLwP - see lines 87-93.

4. In the methods section. If the 15000 Facebook members, how many were interested in taking part in the study? Of these, how many did not meet the inclusion criteria?

Response: 17 expressed interest, 10 participants consented to take part in the study, all 10 met the inclusion criteria, 1 participant dropped out (without providing an explanation) leaving a total sample size of 9. This has been more explicitly explained in the manuscript - see lines 163-164.

5. In terms of inclusion criteria, no maximum age limit was set for participation. How do you estimate the sample size?

Response: The sample size for this study was determined based on the qualitative nature of the research, and the aim of gaining in-depth insights into participants' experiences. We aimed for a size that focused on achieving data saturation, which was indicated when no new themes emerged as we conducted interviews (i.e., new information from the interviews no longer contributed to the research question). Data saturation was achieved confirming that our sample size was adequate for capturing the nuances of participants' perspectives. This has been reflected in the methods of the paper, see lines 166-168.

6.As the "topic guides" that you used in the interviews, could you provide them? At least the headings.

Response: Yes, these have now been provided as supplementary materials for this submission.

7. In the results section: In the first key theme you provide the frequency of participants who believed, who reported… but not in the rest of the themes. Please provide the number of participants who responds each idea or issue.

Response: These have been added throughout the results.

8. At the end you mention that no difference was observed by age groups, severities or sex, but what about the psoriasis duration?

Response: There did not seem to be any difference for this either. This is reflected in the results, see lines 417 - 418.

9. General comments: Please check carefully abbreviations. For example, at the end of the second paragraph of the introduction you did not use the abbreviation for quality of life, or in the third paragraph of the methods section you did not use the abbreviation for people living with psoriasis.

Response: These corrections have been made, and this has been checked throughout the submission

Reviewer: 3
Ms. María Asunción Ballester-Martinez, Hospital Universitario Ramon y Cajal
Comments to the Author:

The authors would like to thank the reviewer for their comments and feedback, which have been addressed below.

1. This is an interesting article regarding dietary modifications in patients with psoriasis. Although exploratory, it includes a very low number of participants, probably not representative of people living with psoriasis (mostly women, 25-53 years, all white; education level not explained; none with conventional systemic treatment (although during the interviews methotrexate is mentioned several times); unknown history of comorbidities or previous dietary interventions; unexplained previous or normal dietary habits; all recruited through Facebook...) Those aspects are partially explained as limitations, but they make the study less generalisable. Besides, the content of the interviews is not sufficiently explained.

Response: For an initial qualitative exploratory study like this, a smaller sample enabled us to explore in-depth the nuances of each participant's perspective, which was essential for uncovering meaningful insights in an understudied area. Whilst smaller, the sample achieved enabled us to achieve our aim of gaining in-depth insights into an area with very limited prior research, particularly in the UK, where no studies have explored this in people living with psoriasis. Qualitative research prioritises in-depth understanding of a phenomenon over generalisability, allowing for a detailed exploration of participants' experiences. Important in an area that is understudied. Additionally, data saturation was achieved, meaning no new themes or insights were found, thereby reinforcing the adequacy of the sample size for this study. These findings serve as a foundation for further exploration and can inform subsequent quantitative studies involving larger, more representative populations within this field. The limitations have been expanded to more explicitly reflect those highlighted in your comment regarding the restricted demographic. See lines 517-521, 526. Additionally, the topic guide has been provided as supplementary material for this manuscript, to provide sufficient explanation and insight into the content of the interviews.

2. It is indicated that they are semi-structured interviews, but the specific content is not specified, which makes the study difficult to reproduce. Supplementary surveys asking about specific data on dietary habits and modifications would be of use to make the study more complete.

Response: The topic guide has been provided as supplementary material for this manuscript, to provide sufficient explanation and insight into the content of the interviews.

It could be also interesting to analyse data taking into consideration if the participant had other comorbidities, which can also have an impact on patient´s diet. This aspect has not been addressed either. As for the results, only some objective results are provided.

Response: The authors agree that this is of value to explore. We have since conducted a larger survey in UK adults where we have collected this information. The findings of this study informed the larger study.

Although the direct quotations are interesting, they do not provide information that is as relevant for the reader. A larger study including more patients with more diverse profiles and quantitative aspects could make the study more complete.

Response: The aim of this study was to gain in-depth insights into an area with very limited prior research, particularly in the UK, where no studies have explored this in people living

with psoriasis. Qualitative research prioritises in-depth understanding of a phenomenon over generalisability, allowing for a detailed exploration of participants' experiences. The quotations provide in-depth and novel insights into the use of diet, and the experiences of PLwP in the UK, which has not been explored before. This is the nature of qualitative research – to provide in-depth insights to inform further studies. We have since conducted a larger survey in UK adults; the survey was informed by the findings of this study.

Reviewer: 4
Dr. Charlayne Mitchell, Northwestern University
Comments to the Author:

Overall, it is a nicely written manuscript. I did have three suggestions first if you decide to increase the size of the study for diversity you should read Hagaman and Wutich, "How Many Interviews Are Enough to Identify Metathemes in Multisited and Cross-cultural Research? Another Perspective on Guest, Bunce, and Johnson's (2006) Landmark Study."

Response: Thank you for this suggestion. The purpose of this study was to explore in-depth the nuances of each participant's perspective, which was essential for uncovering meaningful insights in an understudied area. Saturation was reached in this study, suggesting that the sample size was adequate. Thank you for sharing this publication, which we are keen to utilise in our future work.

Second, I wanted to ask if, on page 6, line 6 under the "method" section "The Consolidated criteria for reporting qualitative research (COREQ) should be capitalized as follows: Consolidated Criteria for Reporting Qualitative Research.

Response: Yes, we have changed this in line with your suggestion.

Third, I am not sure if there is a transcription error or if Participant 1 misspelled "red meat" as "read meat" in the quote inserted on line 6 page 14 under "Key Theme 3: Dietary information."

Response: Yes, this was a spelling mistake, thank you for spotting this. It has now been corrected in line with the above suggestion and have checked throughout the manuscript too.
